# Treatment of endo-periodontal lesion using leukocyte-platelet-rich fibrin. A case report

**DOI:** 10.25100/cm.v43i4.2140

**Published:** 2017-12-30

**Authors:** Pablo Betancourt, Ricardo Elgueta, Ramon Fuentes

**Affiliations:** 1 Research Centre in Dental Sciences (CICO), Facultad de Odontología, Universidad de La Frontera, Temuco, Chile; 2 Facultad de Odontologia , Universidad Mayor, Santiago de Chile, Chile

**Keywords:** Blood platelets, leukocytes, fibrin, surgical procedures, endodontics, Plaquetas sanguíneas, leucocitos, fibrina, procedimientos quirúrgicos, endodoncia

## Abstract

**Case Description:**

The main objective of this paper was to report the clinical effectiveness of leukocyte- platelet- rich fibrin (L-PRF) in the treatment of a combined endo-periodontal lesion of an upper first premolar.

**Clinical Findings:**

The tooth had a profound abfraction on the vestibular aspect and presented no mobility but revealed a deep pocket measuring of 11 mm on the mesial vestibular aspect and 14 mm on the mesial palatine aspect. The three dimensional image analysis showed total bone loss in the mesial aspect and an extensively bone loss of the vestibular aspect of the vestibular root.

**Treatment and Outcome:**

Endodontic treatment was performed and periodontal access surgery (surgical periodontal therapy) was done with the application of autologous L-PRF. Three month and 6 months after surgery, the cone beam computed tomography (CBCT) exams showed no bone regeneration in any aspect of the tooth. However, periodontal examination showed a significative improvement in the deepness of surcus. The mesial vestibular aspect had a deep pocket of 3 mm and 5 mm on the mesial palatine aspect showing a reduction in deepness of 8 mm and 9 mm, respectively.

**Clinical Relevance:**

The actual treatment for teeth with bad prognosis is the extraction and replacement with implants. Even though implants are capable of restore function and aesthetic, the abuse of this approach have led to the loss of teeth that could be successfully treated with a less invasive technique. The prognosis of teeth with endoperiodontal lesion is poor but could be enhanced with regenerative therapies. Until now there are no clinical trials and just four case report about the treatment of these teeth with platelet rich fibrin.

## Resumen

**Descripción del caso:**

El objetivo de este artículo fue reportar la efectividad clínica del uso de fibrina rica en plaquetas y leucocitos (L-PRF) en el tratamiento de una lesión endoperiodontal en un primer premolar superior.

**Hallazgos clínicos:**

El diente presentaba una abfracción profunda en la superficie vestibular, sin movilidad, pero reveló una bolsa profunda de 11 mm en la superficie mesio-vestibular y de 14 mm en la superficie palato-mesial. El análisis tridimensional de la imagen mostró una pérdida ósea total en la superficie mesial y una pérdida ósea extensa de la cara vestibular de la raíz.

**Tratamiento y resultado:**

Se realizó el tratamiento endodóntico y posteriormente cirugía de acceso periodontal (terapia periodontal quirúrgica) con la aplicación de L-PRF autóloga. Tres meses y 6 meses después de la cirugía, los examenes de Tomografía Computarizada Cone Beam (CBCT) no evidenciaron regeneración ósea en ningún sector del diente. Sin embargo, el examen clínico periodontal mostró una mejora significativa en la profundidad de la bolsa. La superficie mesio-vestibular registró una profundidad de bolsa de 3 mm y 5 mm en la superficie mesio-palatino, mostrando una reducción en la profundidad de 8 mm y 9 mm, respectivamente.

**Relevancia clínica:**

El tratamiento actual para los dientes con mal pronóstico es la extracción y el reemplazo con implantes. A pesar de que los implantes son capaces de restaurar función y estética, el abuso de este enfoque ha llevado a la pérdida de dientes que podrían tratarse con éxito con una terapia más conservadora. El pronóstico de los dientes con lesión endoperiodontal es pobre, pero podría mejorarse con el uso de terapias regenerativas. Hasta ahora no hay ensayos clínicos y solo cuatro reportes de casos acerca del tratamiento de estos dientes con fibrina rica en plaquetas y leucocitos.

## Introduction

The periodontium and the pulp are closely related, having embryonic, anatomic and functional interrelationship. The main anatomic pathways by which the pulp and periodontal ligament communicate are dentinal tubules, lateral and accessory canals and the apical foramen ^1^. Besides, palatogingival groove, root perforations and vertical root fractures have been described as communicative pathways. These pathways allow the exchange of bacteria and inflammatory bio-products between the pulp and the periodontal ligament. Thus, a primary periodontal disease can cause a degenerative process in the pulp and, in the same way, an intrapulpal infection can degenerate the periodontium ^2^.

The treatment of endo-periodontal lesion depends on the diagnosis and differentiating between endodontic and periodontal disease ^3^. Once the correct diagnosis is established and the lesion is classified correctly, treatment is indicated, and it may consist in pure endodontic therapy, pure periodontal therapy, or both ^3^. On the other hand, the prognosis of these lesions depends on the structures involved. When there is an extensively loss of attachment the prognosis of the tooth is generally poor, but it can be improved with bone grafting and guided tissue regeneration. Recently, the use of blood derivate products, such as leukocyte- platelet- rich fibrin have been use to accelerate and improve the healing process of the periodontal tissue involved in endo-periodontal lesions ^4^.

Leukocyte- platelet- rich fibrin (L-PRF) is an autologous concentrate obtained through the centrifugation of blood of the same patient in which it will be used. In this concentrate most of the platelets, leucocytes, growth factors and cytokines are contained in a strong fibrin matrix. The fibrin matrix influences the biology of the material and the cells trapped inside ^5^, allow the slow liberation of molecules, and therefore has a relatively long-term effect. Moreover, fibrin itself has a strong general influence on the healing processes ^6^, particularly through the promotion of angiogenesis ^7^. Clinical studies have shown that L-PRF used alone (without bone graft) enhances bone regeneration in 3-wall intra bony defects ^8^. However, there are very few studies aimed to evaluate the clinical effect of L-PRF on endo-periodontal lesions. 

The main objective of this paper was to report the clinical effectiveness of leukocyte- platelet- rich fibrin in the treatment of a combined endo-periodontal lesion.

## Case report

This research was conducted in concordance with Helsinki Declaration and patient signed the informed consent.

### Social and dental history

 A 52 year's old male patient was derivate to the Dental Clinic of the Universidad de La Frontera, for the endodontic treatment of the upper left first premolar. Clinically the tooth had a profound abfraction on the vestibular aspect, in close proximity with the root canal. The tooth had no mobility but revealed a deep pocket measuring of 11 mm on the mesial vestibular aspect and 11 mm on the mesial palatine aspect. There were no other periodontal involved teeth in the remaining dentition. The patient was a smoker (6/day) and had no systemic diseases associated.

**Figure 1:**
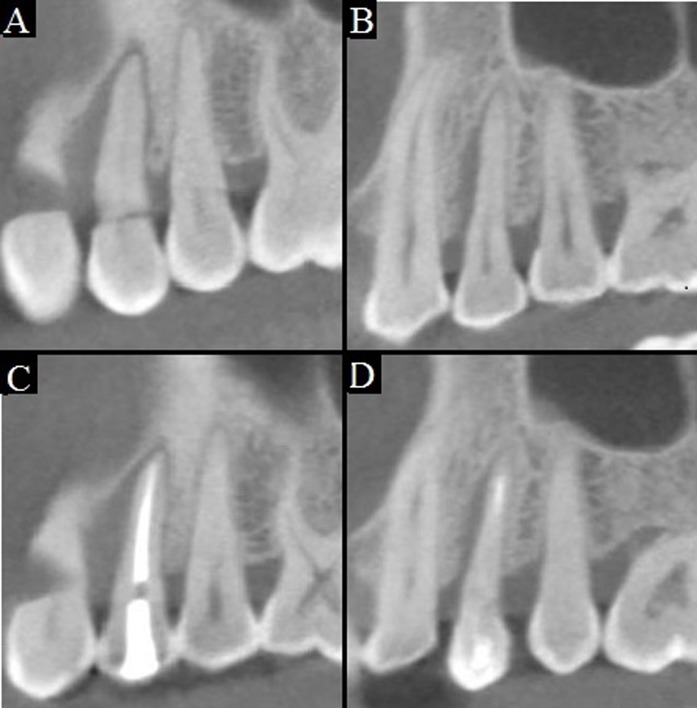
A-B: CBCT images of the vestibular and palatal root previous to the surgical treatment. C-D: CBCT images of the vestibular and palatal root 6 month after the surgical treatment, showing no bone gain.

The three dimensional image analyses were realized by Cone Beam Computed Tomography (CBCT). The tooth in question showed total bone loss in the mesial aspect of the vestibular root and an extensively bone loss of the vestibular aspect of the vestibular root. The palatine root had bone loss involvement in the mesial aspect (Fig. 1 A-B).

### Endodontic treatment

At clinical examination, was observed that tooth 2.4 was asymptomatic, without any endodontic treatment started, and a slight discoloration. Vitality tests were negative. The tooth present two root and there was observed no bleeding at the endodontic access. 

In the treatment stage, initially the two root canals were permeabilized with #10 and #15 K-files (Dentsply Maillefer, Ballaigues Switzerland). The working length was determined with a periapical radiograph and verified with the aid of an electronic apex locator (Root ZX®, J. Morita, Japan). After this, the canals shaping was performed by a rotatory reciprocating files (WaveOne®, Dentsply Maillefer, Ballaigues, Switzerland) at 350 rpm, following the manufacturers indication, with a Crown-down technique. This system used an only single file Primary 025/08 in all canals. In the entire canal shaping stage, was perfused with sodium hypochlorite at 2.5% and permeabilized with #15 K-file to remove the smear layer of the root canals. The canal obturation was realized with WaveOne® points with the same taper of the Primary file, achieving a proper fit in the apex. As a final root canal sealer, TopSeal® (Dentsply Maillefer, Ballaigues Switzerland) was used. Finally a radiographical and clinical control was done at 7 and 14 days after the treatment, observing no clinical symptoms or radiographical findings of treatment failure.

### Periodontal and regenerative treatment

The periodontal surgery was made two week after the endodontic treatment. 

The patient was informed about the imperative necessity of stop smoking and 1 hour prior the surgery he took Amoxicilin 875 mg (Optamox®, Pharma Investi, Chile) and Ketoprofen 200 mg (Forenol Lp®, Pharma Investi, Chile) and mouth rinse with chlorhexidine 0.12% (Oralgene®, Maver, Chile).

**Figure 2:**
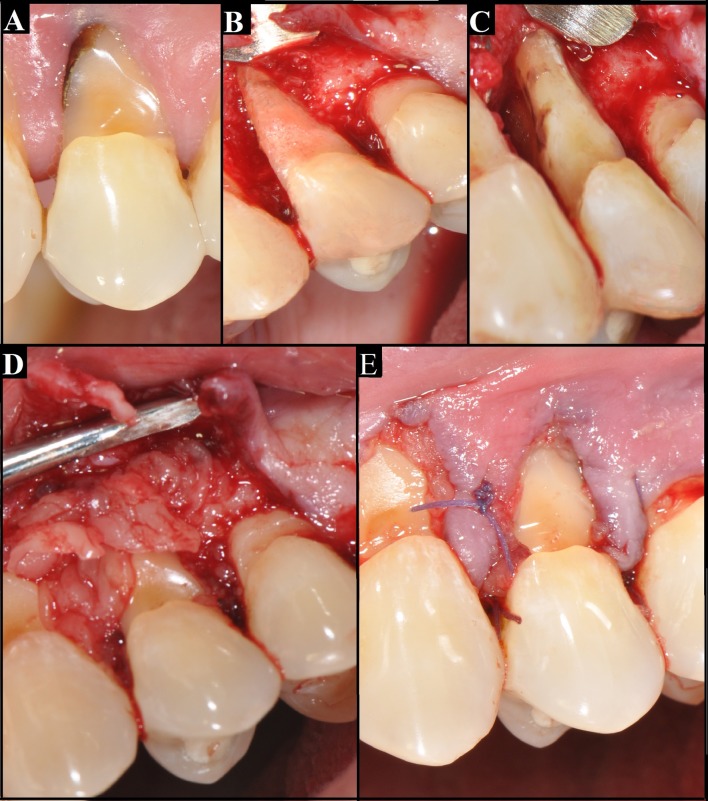
A. previous state of the tooth, showing a deep abfraction. B: vestibular aspect, after raising the flap, showing a big bone resorption. C: mesial aspect of roots, it can be observed the furcation involvement. D: Leucocyte - platelet - fibrin concentrate covering the roots. E: Flap suture.

The surgical area was anesthetized with lidocaine 2%. Sulcular incision around the involved tooth, followed by a releasing vertical incision on the mesial side, was made with 15 scalpel blade (HuFriedy®, Leimen, Germany). A full flap was raise, but minimally extended on the distal side (Fig. 2 B). After recognize the site of interest, a total root planning was made with a 5-6 Gracey curette (HuFriedy®, Leimen, Germany) removing granulation tissue and calculus/detritus (Fig. 2C). The surface was conditioned with a 250 mg of tetracycline (Laboratorio Chile, Chile) diluted in 1 cc of serum by 1 min. The procedure was made with profusely irrigation with saline. To obtain the clot, the blood sample was obtained from an antecubital vein, with a butterfly (No. 23 G, Blood Collection Set + Luer Adapter, Vacuette®, Austria) and plastic shirt (BD Vacutainer®, USA). Blood samples were dispensed into 6 blood containing tubes of 9 mL each (Z Serum Clot Activator: Vacuette®, Kremsmunster, Austria), and were immediately centrifuged (Labo- fuge® 300; Heraus GmbH, Hanau, Germany) at 2,700 rpm for 12 min. The centrifuged blood mass presented with a structured fibrin clot in the middle of the tube, between the acellular plasma on top and red corpuscle layer on the bottom. After centrifugation, the L-PRF clot was removed from the tube using sterile tweezers, separated from the red blood cell (RBC) base using scissors, and placed in a sterile metal cup. Each L-PRF clot started to release its serum (L-PRF-clot exudate) and was ready for compression into the membrane. 

Once the roots and the furcation were clean, fibrin clots were placed in bone defect, one on top of the other and in opposite directions, and gently placed on the vestibular aspect, covering the root (Fig. 2D). Closure of soft tissue flap was done with polyglactin sutures 5-0 (Vicryl(tm), Ethicon, New Jersey, USA) (Fig. 2 E). Any complication on surgery was observed. Indication of continue pharmacologic treatment and mouth rinse with chlorexidine 0.12% (Oralgene®, Maver, Chile) for 1 week were indicated. The sutures were removed 15 days after surgery. The patient was scheduled for regular recall at 7, 15, 90 and 180 days.

**Figure 3:**
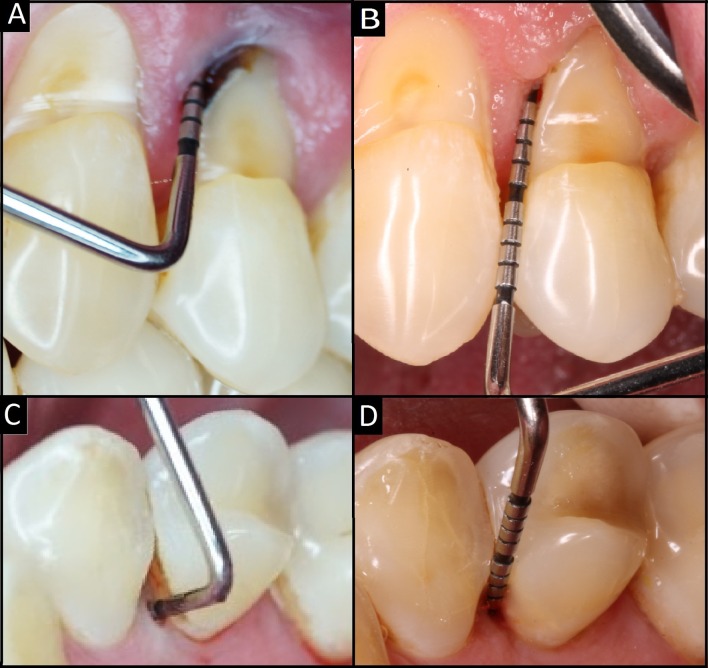
A-C: Pocket depth prior surgery; 11 mm and 14 mm on the mesial vestibular and mesial palatine aspect. B-D: Pocket depth three month after surgery; 3 mm and 5 mm on the mesial vestibular and palatine aspect.

Three month and 6 months after surgery, the CBCT exams showed no bone regeneration in any aspect of the tooth (Fig.1 C-D). However, periodontal examination shows a significative improvement in the deepness of surcus. The mesial vestibular aspect had a deep pocket of 3 mm and 5 mm on the mesial palatine aspect showing a reduction in deepness of 8 mm and 9 mm, respectively (Fig. 3 B-D).

## Discussion

The actual treatment for teeth with bad prognosis is the extraction and replacement with implants. Even though implants are capable of restore aesthetic and function, the abuse of this approach have led to the loss of teeth that could be successfully treated with a less invasive technique, and still be functionally useful. In this case report, an upper premolar with a severe endo-periodontal lesion was successfully treated with a conservative treatment.

The prognosis of a true combined endoperiodontal lesion is often poor or even hopeless, especially when periodontal lesions, like in this case report, compromises an extensive loss of attachment ^2^. However, with the advent of new regenerative procedures, successful rate of endo-periodontal lesions could be enhanced.

Leukocytes and platelet rich fibrin is a second generation of platelet concentrated in which most of the platelets and leucocytes are contained within a strong fibrin matrix ^9^ ^,^ ^10^. The L-PRF clot contains more than 50% of the leukocytes from the initial blood harvest ^11^. The fibrin matrix allows the slow liberation of molecules because the concentrate does not dissolve quickly and the fibrin matrix is slowly remodelled, thus obtaining a relatively long-term effect ^12^. Moreover, fibrin itself promotes angiogenesis ^7^.

In vivo studies have shown that L-PRF promotes periodontal regeneration and enhances alveolar bone augmentation ^13^. *In vitro* studies have shown that L-PRF stimulates the proliferation of fibroblasts, periodontal progenitor cells and osteoblasts ^10^ ^,^ ^13^. L-PRF is also reported to promote the differentiation of osteoblasts and protein production ^14^. Additionally, a wide range of studies have shown significant benefits from the presence of leukocytes in the L-PRF. Leukocytes have a role in cleaning up the surgical site, regulating the expression of inflammatory cytokines and inflammatory mediators ^5^ ^,^ ^10^, they have an anti-infectious impact ^5^ ^,^ ^15^ ^-^ ^17^, and also secrete a battery of growth factors ^5^, one of which is vascular endothelial growth factor which stimulates angiogenesis. All these biological characteristic of the L-PRF could enhance the probability of clinical success of teeth with severe bone loss and periodontal attachment.

In this case report was observed a gain in the clinical attachment level and reduction in probing depth. However we could not observe any bone gain at any aspect of the tooth. Karunakar *et al*. ^18^, observed adequate radiographic bone fill in two teeth with endoperiodontal lesions, treated with L-PRF and same results were observed by Singh ^19^ with the use of platelet-rich plasma (PRP). However, it is important to notice that these cases previously mentioned, did not used the platelet concentrate alone but with the adjacent of a bone graft material, that could be responsible for the bone gain.

It is questionable if the reduction in probing deep obtained in this study would be different if there were no use of a platelet concentrate. In despite of the potential positives effects of the L-PRF very few studies had tempted to prove its clinical and radiographic effectiveness in the treatment of endoperiodontal lesions ^4^ ^,^ ^18^ ^,^ ^20^ ^,^ ^21^. In the literature we do not found clinical trials tending to solve the question whether the L-PRF enhance the probability of success of the periodontal treatment, in combined endo-periodontal lesions. Therefore it is important to conduct well design studies that solve this matter.
