# Supplementary material for: Treatment of endo-periodontal lesion using leukocyte-platelet-rich fibrin. A case report
Source: Colomb Med (Cali). 2017 Dec 30;48(4):204–7. doi: 10.25100/cm.v43i4.2140 (PMC5896727; doi:10.25100/cm.v43i4.2140)
Supplement: Related texts

# Case report: Tratamiento de una lesión endoperiodontal utilizando fibrina rica en plaquetas y leucocitos. Un reporte de caso

## Introducción

El periodonto y la pulpa están estrechamente relacionados, a través de una interrelación embrionaria, anatómica y funcional. Las principales vías anatómicas por las que se comunican la pulpa y el ligamento periodontal son los túbulos dentinarios, los canales laterales y accesorios y el foramen apical ^1^^).^ Además, el surco palatogingival, las perforaciones radiculares y las fracturas radiculares verticales se describen como vías comunicativas. Estas vías permiten el intercambio de bacterias y bioproductos inflamatorios entre la pulpa y el ligamento periodontal. Por lo tanto, una enfermedad periodontal primaria puede causar un proceso degenerativo en la pulpa y, de la misma manera, una infección intrapulpar puede degenerar el periodonto ^(^^2^^).^

El tratamiento de la lesión endoperiodontal depende del diagnóstico y de la diferenciación entre enfermedad endodóntica y periodontal ^3^. Una vez que se determina el diagnóstico correcto y la lesión se clasifica correctamente, se señala cuál es el tratamiento y éste puede consistir en terapia endodóntica, terapia periodontal o ambas ^3^. Por otro lado, el pronóstico de estas lesiones depende de las estructuras involucradas. Cuando hay una pérdida extensa de inserción, el pronóstico del diente es generalmente pobre, pero se puede mejorar con injertos óseos y regeneración guiada de tejidos. Recientemente, el uso de productos derivados de la sangre, como la fibrina rica en plaquetas y leucocitos (L-PRF) se ha utilizado para acelerar y mejorar el proceso de curación del tejido periodontal implicado en las lesiones endoperiodontales ^4^^).^

La fibrina rica en plaquetas y leucocitos es un concentrado autólogo obtenido mediante la centrifugación de sangre del mismo paciente en el que se usará. En este concentrado, la mayoría de las plaquetas, leucocitos, factores de crecimiento y citoquinas están contenidas en una matriz de fibrina sólida. La matriz de fibrina influye en la biología del material y en las células contenidas en su interior ^5^, además, permite la lenta liberación de las moléculas y, por lo tanto, tiene un efecto relativo a largo plazo. Así mismo, la fibrina en sí misma tiene una fuerte influencia en los procesos de curación^6^, particularmente a través de la promoción de la angiogénesis^7^. Los estudios clínicos demuestran que la L-PRF utilizada sola (sin injerto óseo) mejora la regeneración ósea en defectos intraóseos de 3 paredes ^8^. Sin embargo, existen muy pocos estudios destinados a evaluar el efecto clínico de L-PRF en las lesiones endoperiodontales.

El objetivo principal de este trabajo fue informar la efectividad clínica de la fibrina rica en plaquetas y leucocitos en el tratamiento de una lesión endoperiodontal combinada.

## Reporte de caso

Esta investigación se realizó en base a la Declaración de Helsinki y el paciente en cuestión firmó el consentimiento informado.

### Historia social y dental

 Un paciente masculino de 52 años fue derivado a la Clínica Dental de la Universidad de La Frontera para el tratamiento endodóntico del primer premolar superior izquierdo. Clínicamente, el diente tenía una profunda abfracción en la superficie vestibular, muy cerca del canal radicular. El diente no tenía movilidad, pero reveló una bolsa profunda que medía 11 mm en la superficie mesio vestibular y 11 mm en la superficie palato mesial. No hubo otros dientes periodontalmente comprometidos en la dentición restante. Paciente fumador (6/día) y no presentaba enfermedades sistémicas asociadas.

El análisis de imagen tridimensional se realizó mediante la tomografía computarizada Cone Beam (CBCT). El diente en cuestión mostró una pérdida ósea total en la superficie mesial de la raíz vestibular y una pérdida ósea extensa de la superficie vestibular de la raíz vestibular. La raíz palatina presentaba pérdida ósea en la superficie mesial (Fig. 1 A-B).

**Figura 1:** A-B: Imágenes CBCT de la raíz vestibular y palatina previas al tratamiento quirúrgico. C-D: Imágenes CBCT de la raíz vestibular y palatina 6 meses después del tratamiento quirúrgico, que no muestran ganancia ósea.

### Tratamiento endodóntico

Al examen clínico, se observó que el diente 2.4 no presentaba síntomas, sin que se iniciara ningún tratamiento endodóntico; sin embargo, presentaba una ligera decoloración. Las pruebas de vitalidad fueron negativas. El diente presentó dos raíces y no se observó sangrado al realizar el acceso endodóntico.

En la etapa de tratamiento, inicialmente los dos canales radiculares se permeabilizaron con limas K #10 y #15 (Dentsply Maillefer, Ballaigues Suiza). La longitud de trabajo se determinó con una radiografía periapical y se verificó con la ayuda de un localizador electrónico apical (Root ZX(r), J. Morita, Japón). Después de esto, la conformación de los canales se realizó mediante limas rotatorias reciprocantes (WaveOne(r), Dentsply Maillefer, Ballaigues, Suiza) a 350 rpm, siguiendo la indicación de los fabricantes, mediante una técnica de Crown-down. Este sistema utilizó sólo la lima primary 025/08 en todos los canales. En toda la etapa de conformación del canal, se irrigó con hipoclorito de sodio al 2,5% y se permeabilizó con un lima K #15 para eliminar la capa de residuos de los canales radiculares. La obturación del canal se realizó con puntas WaveOne® con la misma conicidad de la lima primary, logrando un ajuste adecuado a nivel apical. Como sellador del canal radicular, se utilizó TopSeal(r) (Dentsply Maillefer, Ballaigues Suiza). Finalmente, se realizó un control radiográfico y clínico a los 7 y 14 días después del tratamiento, sin observar síntomas clínicos ni hallazgos radiográficos relacionados con algún fracaso del tratamiento.

### Tratamiento periodontal y regenerativo

La cirugía periodontal se realizó dos semanas después del tratamiento endodóntico.

El paciente fue informado sobre la necesidad de dejar de fumar y 1 hora antes de la cirugía tomó Amoxicilina de 875 mg (Optamox®, Pharma Investi, Chile) y Ketoprofeno 200 mg (Forenol Lp®, Pharma Investi, Chile) y se aplicó enjuague bucal con clorhexidina 0.12% (Oralgene®, Maver, Chile).

El área quirúrgica se anestesió con lidocaína al 2%. Se realizó una incisión sulcular alrededor del diente involucrado, seguida de una incisión de descarga vertical en el lado mesial, con una hoja de bisturí tipo 15 (HuFriedy®, Leimen , Alemania). Se levantó un colgajo de espesor total, pero se extendió mínimamente en el lado distal (Fig. 2 B). Después de reconocer el sitio de interés, se realizó un curetaje radicular total con una cureta Gracey 5-6 (HuFriedy(r), Leimen, Alemania) que eliminó el tejido de granulación y el cálculo/détritus (Fig. 2C). La superficie se acondicionó con 250 mg de tetraciclina (Laboratorio Chile, Chile) diluida en 1 cc de suero por 1 min. El procedimiento se hizo con irrigación profusa con solución salina. Para obtener el coágulo, la muestra de sangre se obtuvo de una vena antecubital, con una jeringa mariposa (No. 23 G, Blood Collection Set + Luer Adapter, Vacuette®, Austria) y camisa plástica (BD Vacutainer®, EE. UU). Se dispensaron muestras de sangre en 6 tubos de 9 ml cada uno (Z Serum Clot Activator: Vacuette(r), Kremsmunster, Austria), y se centrifugaron inmediatamente (Labo- fuge® 300; Heraus GmbH, Hanau, Alemania) a 2,700 rpm durante 12 min. La sangre centrifugada se presentó como un coágulo de fibrina estructurado en el medio del tubo, entre el plasma acelular en la parte superior y la capa del corpúsculo rojo en la parte inferior. Después de la centrifugación, el coágulo de L-PRF se retiró del tubo usando pinzas estériles, se separó de la base de las células rojas sanguíneas (RBC) con tijeras y se colocó en una copa de metal estéril. Cada coágulo de L-PRF comenzó a liberar su suero (exudado de coágulos de L-PRF) y demostró estar preparado para la compresión en la membrana.

**Figura 2:** A. Aspecto inicial del diente, que muestra una abfracción profunda. B. Superficie vestibular, después de levantar el colgajo, mostrando una gran reabsorción ósea C. Superficie mesial de las raíces, se puede observar la afectación de la furcación. D. Concentrado de fibrina rica en plaquetas y leucocitos cubriendo las raíces. E.- Sutura del colgajo.

Una vez que las raíces y la furcación estuvieron limpias, se colocaron coágulos de fibrina en el defecto óseo, uno encima del otro y en direcciones opuestas, y se colocaron suavemente en la superficie vestibular, cubriendo la raíz (Fig. 2D). El cierre del colgajo de tejido blando se realizó con suturas de poliglactina 5-0 (Vicryl (tm), Ethicon, Nueva Jersey, EE.UU.) (Fig. 2 E). No se observó complicación alguna en la cirugía. Se indicó continuar el tratamiento farmacológico y el enjuague bucal con Clorhexidina al 0.12% (Oralgene®, Maver, Chile) durante una semana. Las suturas se retiraron 15 días después de la cirugía. El paciente fue programado para un control regular a los 7, 15, 90 y 180 días.

Tres meses y 6 meses después de la cirugía, los exámenes CBCT no mostraron regeneración ósea en ningún aspecto del diente (Fig. 1 C-D). Sin embargo, el examen periodontal mostró una mejora significativa en la profundidad de la bolsa. La superficie mesio vestibular registró una profundidad de bolsa de 3 mm y 5 mm en la superficie mesio palatina, mostrando una reducción en la profundidad de 8 mm y 9 mm, respectivamente (Fig. 3 B-D).

**Figura 3:** A-C: Profundidad de la bolsa periodontal previo a la cirugía; 11 mm y 14 mm en la superficie mesio vestibular y mesio palatina. B-D: Profundidad de la bolsa periodontal 3 meses después de la cirugía; 3 mm y 5 mm en la superficie mesio vestibular y mesio palatina.

## Discusión

El tratamiento actual para los dientes con mal pronóstico es la extracción y el reemplazo con implantes. A pesar de que los implantes son capaces de restaurar la estética y la función, el abuso de éstos ha llevado a la pérdida de dientes que no presentarían problema con una técnica menos invasiva, permaneciendo funcionalmente útiles. En este caso, un premolar superior con una lesión endoperiodontal severa se trató satisfactoriamente con un tratamiento conservador.

El pronóstico de una lesión combinada endoperiodontal verdadera a menudo es pobre, especialmente cuando las lesiones periodontales, como en este caso, comprometen una pérdida extensa de la inserción ^2^. Sin embargo, con el advenimiento de nuevos procedimientos regenerativos, la tasa exitosa de lesiones endoperiodontales podría mejorarse.

La fibrina rica en plaquetas y leucocitos es una segunda generación de plaquetas concentradas en la que la mayoría de las plaquetas y leucocitos están contenidos dentro de una fuerte matriz de fibrina ^9^^,^^10^. El coágulo L-PRF contiene más del 50% de los leucocitos de la extracción de sangre inicial ^11^. La matriz de fibrina permite la lenta liberación de moléculas porque el concentrado no se disuelve rápidamente y la matriz de fibrina se remodela lentamente, obteniendo así un efecto a largo plazo ^12^. Además, la fibrina por sí misma promueve la angiogénesis ^7^.

Los estudios *in vivo* han demostrado que L-PRF promueve la regeneración periodontal y mejora el aumento óseo alveolar ^13^. Los estudios *in vitro* demuestran que L-PRF estimula la proliferación de fibroblastos, las células progenitoras periodontales y los osteoblastos ^(10, 13)^_._ También se informa que L-PRF promueve la diferenciación de los osteoblastos y la producción de proteínas ^14^. Además, una amplia gama de estudios demuestra beneficios significativos de la presencia de leucocitos en la L-PRF. Los leucocitos tienen un rol importante en la limpieza del sitio quirúrgico, regulan la expresión de citoquinas inflamatorias y mediadores inflamatorios ^5^^,^^10^, tienen un impacto antiinfeccioso ^5^^,^^15^^-^^17^ y también secretan una batería de factores de crecimiento ^5^, uno de los cuales es el factor de crecimiento endotelial vascular que estimula la angiogénesis. Todas estas características biológicas de L-PRF podrían aumentar la probabilidad de éxito clínico de los dientes con pérdida ósea severa e inserción periodontal.

En este caso, se observó una ganancia en el nivel de inserción clínica y una reducción en la profundidad del sondaje. Sin embargo, no se pudo observar ninguna ganancia ósea en ninguna superficie del diente. Karunakar et al. ^18^, observaron un relleno óseo radiográfico adecuado en dos dientes con lesiones endoperiodontales tratados con L-PRF y los mismos resultados fueron observador por Singh ^19^ con el uso de plasma rico en plaquetas (PRP). Sin embargo, es importante notar que en estos casos mencionados anteriormente, no se usó el concentrado de plaquetas solo, sino con el material de injerto óseo adyacente, que podría ser responsable de la ganancia ósea.

Es cuestionable si la reducción en la profundidad de sondeo obtenida en este estudio sería diferente si no hubiera uso de un concentrado de plaquetas. A pesar de los posibles efectos positivos de la L-PRF, muy pocos estudios habían tenido la intención de demostrar su efectividad clínica y radiográfica en el tratamiento de las lesiones endoperiodontales ^4^^,^^18^^,^^20^^,^^21^. En la literatura, no encontramos ensayos clínicos que tiendan a resolver la pregunta de si la L-PRF aumenta la probabilidad de obtener un tratamiento periodontal exitoso en las lesiones endoperiodontales combinadas. Por lo tanto, es importante realizar estudios bien diseñados que resuelvan ésta interrogante.
